# Determination of the radii of coated and uncoated silicon AFM sharp tips using a height calibration standard grating and a nonlinear regression function

**DOI:** 10.3762/bjnano.14.99

**Published:** 2023-12-15

**Authors:** Perawat Boonpuek, Jonathan Robert Felts

**Affiliations:** 1 School of Manufacturing Engineering, Institute of Engineering, Suranaree University of Technology, 111 University Avenue, Muang, Nakhon Ratchasima 30000, Thailand; 2 Advanced Manufacturing Laboratory, J. Mike Walker ’66 Department of Mechanical Engineering, College of Engineering, Texas A&M University, College Station, Texas, 77843, USA

**Keywords:** AFM tip calibration, nonlinear regression curve fitting

## Abstract

AFM sharp tips are used to characterize nanostructures and quantify the mechanical properties of the materials in several areas of research. The analytical results can show unpredicted errors if we do not know the exact values of the AFM tip radius. There are many techniques of in situ measurements for determining the actual AFM tip radius, but they are limited to uncoated tips. This paper presents an alternative and simple method to determine the radii of coated tips and an uncoated tip. Pt-coated, Cr/Au-coated, and uncoated Si tips were used to scan a calibration standard grating in AFM contact mode with sub-nanonewton load to obtain the curved scan profile of the edge corner of the grating structure. The data points of the curved profile of each tip were fitted with a nonlinear regression function to estimate the curvature radius of the tip. The results show that the estimated radius of the coated tips is in the range of nominal values provided by the tip manufacturer, while the estimated radius of the uncoated Si tip is bigger than the nominal radius because of tip blunting during the scan. However, this method yields an accurate estimate of the tip radius with a low root mean squared error of the curve fitting results.

## Introduction

Atomic force microscopy (AFM) with a sharp tip is typically used to characterize nanostructured materials, for example, graphene, carbon nanotubes, nanoscale semiconductors, biomaterials, and molecules. Mechanical properties such as surface stiffness, adhesion, friction, electrostatics, and electrowetting can be measured [1–4]. In contact mode scanning, the contact area between the AFM tip and the sample, which depends on the tip radius, defines how accurately the AFM tip determines those properties and the shape of fabricated micro- and nanostructures. The contact radius of the tip is a key variable for calculating the stiffness and Young’s modulus of the material by fitting force curves with contact mechanics models and extracting the adhesion and friction forces [5–6]. If we do not know the exact value of the tip radius, the sample image with the observation of scanning frequency and the calculation results are not accurate. This indicates that the measurement results strongly depend on the geometry of the AFM tip [7–8].

For example, the Pt-coated HQ:NSC18/Pt tip (for electrical force modulation AFM probes) and the Cr/Au-coated HQ:NSC16/Cr-Au tip (for tapping mode AFM probes with long AFM cantilever) produced by MikroMasch [9] have estimated nominal tip radii lower than 30 or 35 nm, respectively. But these are not the true radii of the tips. Therefore, AFM users need to determine the actual tip radius before probing micro- and nanostructures and measuring the mechanical properties of a sample. In previous studies, tip shape and radius at the sharp end were measured using a tip characterizer and tip qualification or standard calibration grating. In one of those studies, the tip geometry was determined by using a well-known sharp-edged silicon structure, which included height patterns with a certified pitch on a nanostructure plate [10]. Three types of AFM tips (silicon nitride, silicon, and high-aspect-ratio tips) were scanned over the calibration pattern. Simultaneously, the AFM measurement signal showed the tip path profile as the real geometry of a fabricated microstructure. The tip radius was obtained from the curvature radius of the curve profile at the top corner of a grating structure (a fabricated square column). Then, the tip radius was estimated by fitting a manually drawn circle to the curve profile of the AFM scan [10]. However, the tip curve profile can differ from the shape of a circle, leading to some deviation of the tip curve profile radius. Another method for tip shape determination is scanning AFM images across a test grating to get a scanline profile of the tip and to compare that with the result of a geometrical model of the tip profile calculated using a tip slope angle. The tip cone angle together with the tip slope angle yield the scanline profile of the tip with its slope angle for tip radius calculation. However, this geometry-based method relies on the tip slope angle, which varies for different types of tips [11]. In noncontact scanning mode, Maragliano et al. showed that a sharper tip (small tip radius) yields a smaller value of free amplitude transition between attractive and repulsive force regimes than a tip with larger radius. The capacitive tip was also used to determine the tip radius through geometric relationship equations. The results of both methods were compared with the nominal values of the tip radius given by manufacturers. Both methods provided a reliable tip radius estimate [12]. However, this approach is not related to the physical aspects of an AFM tip in contact scanning measurements, in which the shape of the tip end needs to be extracted. For non-sharp tips, determining the tip geometry if there is a lack of accurate knowledge about the real geometry of the characterizer pattern is difficult. This is because the AFM scanline profiles of flared or spherical tip ends differ from the actual vertical and sloped sidewalls of the characterizer (i.e., calibration grating) when the tip moves over the corner edges. To achieve an accurate estimation of the radius, a reconstruction method for the tip scan profile has been developed by fitting the measured points to the morphological filter surface of the characterizer [13]. Yet, this reconstruction technique may not be applicable for very sharp tips that can make a smaller difference in the scanned profile offset at the corners of the characterizer.

A method for characterizing blunt tips was also demonstrated. Nanoindentation using four blunt tips on an AFM cantilever was performed in a normal loading process on a soft PVC sheet for 30 nm depth to obtain sufficient data of force curves. The blunted tips were imaged by SEM immediately after running the nanoindentation process in the AFM program. Then, each tip radius was determined by fitting the force loading curves with the Hertz model equation using the indentation depth and the reduced modulus [14]. Real-time measurements of tip radii based on analysis of the power spectral density (PSD) of the topography of a surface of an ultrananocrystalline diamond were carried out to detect changes in tip radius during tapping mode scanning. For each scan, the frequency data in relation to the PSD were collected to observe the tip blunting behavior. The quantitative determination of the tip radii compared to transmission electron microscopy (TEM) images of the tips showed corresponding results between the blunted tips and the PSD curves, concluding that blunter tips produced a higher frequency with low PSD value than sharp tips [8]. However, the aforementioned measurements are theoretical approaches lacking consideration of the measured point-to-point data of the scanline profile in an (*x*, *y*) coordinate system for calculations of the exact tip radius after the scan. In addition, those studies are limited to the use of uncoated AFM tips with reflectively coated cantilevers.

Here, we present the actual measurement of the radius of coated and uncoated AFM tips using the contact scan mode with sub-nanonewton normal load on a height calibration standard grating. The round corner segment of the scanline profile of the tip apex was used to determine the tip radius with a nonlinear regression method. This method fits the arc curve through the measured point-to-point data of tip position in an (*x*, *y*) coordinate system, allowing us to obtain the exact value of the tip radius.

## AFM Tips and Calibration Standard Grating

**Figure 1:**
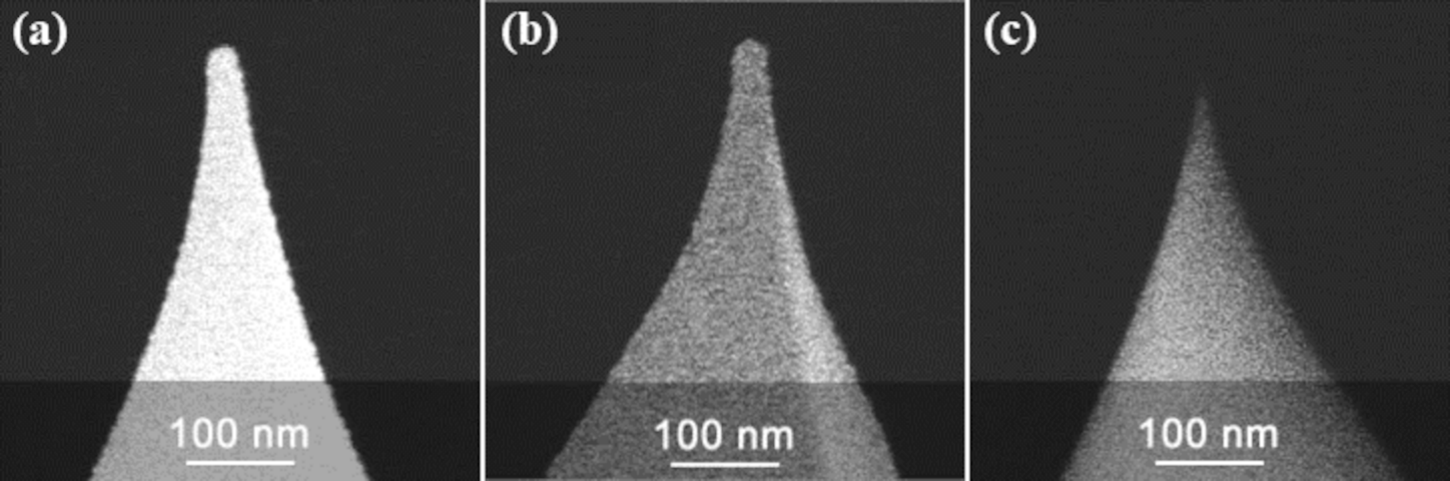
SEM images of (a) the Pt-coated tip, (b) the Cr/Au-coated tip, and (c) the uncoated silicon tip [9].

Three types of AFM sharp tips were used, namely a Pt-coated tip (HQ:NSC18/Pt, nominal radius < 30 nm for electrical, force modulation AFM; Figure 1a), a Cr/Au-coated tip (HQ:NSC16/Cr-Au, nominal radius < 35 nm for tapping mode AFM; Figure 1b), and an uncoated silicon tip (HQ:CSC17/No Al, nominal radius < 8 nm for regular force contact AFM; Figure 1c), all supplied by MikroMasch. The full cone angle for all tips is 40° as given by MikroMasch [9]. The tip end is a hemisphere with specific radius. For the coated tips, the tip radius includes the coating material. Figure 1 shows SEM images of the three different tips.

**Figure 2:**
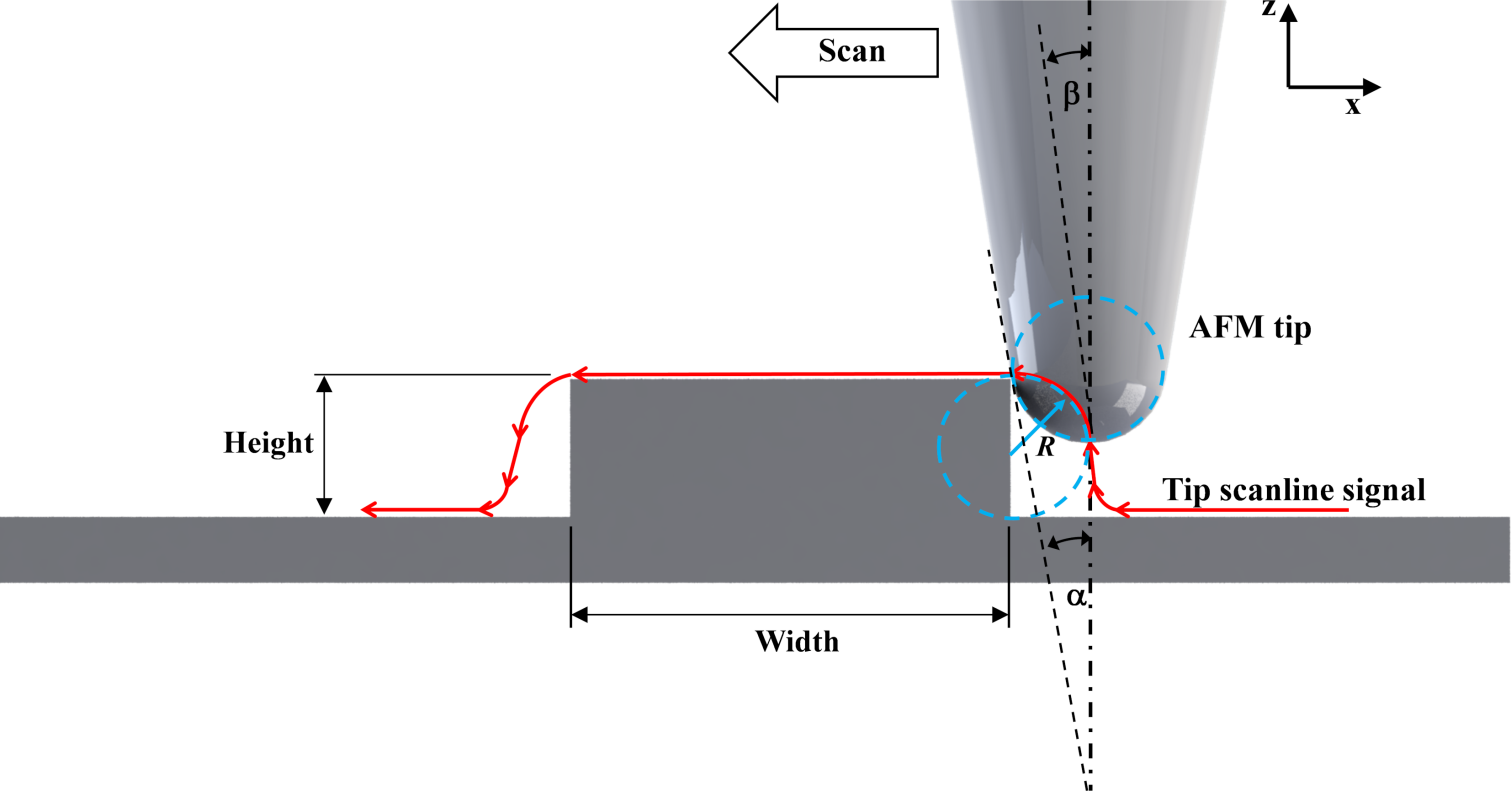
A side-view schematic of the scanned line profile of the tip along a calibration grating.

The calibration standard grating used in this experiment is a HS-20MG height calibration standard (supplied by Budget Sensor) with an external size of 1 mm × 1 mm and an inner size of 500 µm × 500 µm [15]. The rectangular height grate pattern is made of SiO_2_ with height = 20 nm, grate distance = 2 µm, and pitch distance = 5 µm, on top of a silicon substrate, which allows for more space for the AFM tips to sweep along the height grate geometry. The corner edge radius of the grating height is not provided in the specification [15]. However, recent in situ determinations of the tip characterizer shape revealed that the radius of curvature of the Si corner edge of the standard grating is sharper (usually <2 nm [16]) than the tip end. Although we do not know the exact value of the edge radius of the height grate from its specification sheet for this measurement, the tip radius profile can be directly determined by the deflection and position signal of the AFM cantilever during contact between that edge surface and the tip end. Our point of interest for determining the tip radius using this standard grate is that the corner edge of the grate comes in contact with the tip cone (the black inclined dashed line on the left of the cone tip below in Figure 2) when the tip scans towards the height grate until the corner edge follows the curved surface of the tip end. Thus, the contact points between the grate’s corner edge and the tip cone surface determine the profile of the curvature radius of the scanning tip. In our experiment, we used the inner standard grating area with a line grate pitch of 5 µm, because the AFM tip can easily scan through each linewidth within a small scan area of 10 µm × 10 µm.

## Determination of Tip Radii

### Scanning the height calibration grating

Linewidth scanning in contact mode using each of the selected tips with an applied constant normal load of 0.01 nN perpendicular to the calibration grating was carried out at room temperature and 40% humidity. The scanning frequency of the tips was 0.03 Hz. The tips were moved from right to left across two pristine grating structures within a scan size of 10 µm × 10 µm. The calibration grating was cleaned with DI water and dried with nitrogen gas before the experiments. For each tip, we performed a step-to-step linewidth scan pass (without repeating at the same scanline) in contact mode with very light loading (0.01 nN) to prevent the tips from wear. The noise effect was not considered here, because the measurement was conducted inside the hood of a MFP-3D Origin (Asylum Research) with noise filter system.

**Figure 3:**
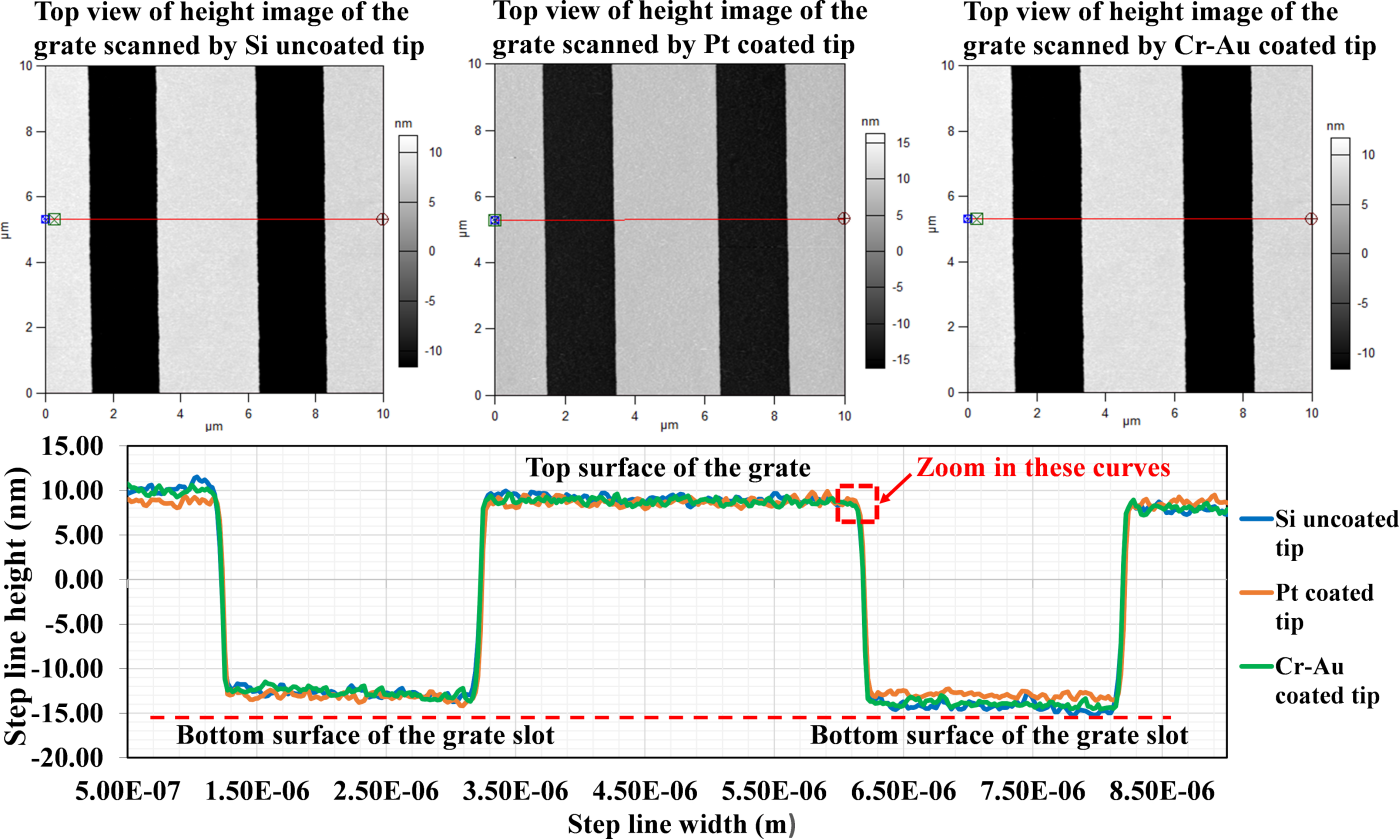
The height images (top view) obtained from scanning results using the three tips (on the upper row) compared to the AFM signal scan profiles of the tips (side view) (on the lower row) extracted from the cross-sectional linewidth (red line).

Considering the geometry of the nanostructure and the scanline signal obtained from the AFM, Figure 2 shows a schematic of the scanning. Since the grating pattern is very small, the height of the calibration structure (20 nm) is obviously about the same level as the tip radius, except for the uncoated Si tip (tip radius < 8 nm). Also, it is steeper than the half-cone angle of the tip, α. So, the steep profile of the AFM scanline signal defined by the steep angle, β, represents the scan path of the apex of the tip sliding along the vertical sidewall of the grate height. Importantly, the curvature profile of the AFM signal scanline at the edge corner of the scanned calibration structure represents the tip radius, *R*, as shown in Figure 2. This analysis of the tip scanline profile is consistent with a previous study [10]. AFM height images from the AFM MFP-3D software were compared for the three different tips (Pt-coated tip, Cr/Au-coated tip, and uncoated Si tip, see more details in Supporting Information File 1, Section S1). The cross-sectional linewidth (red line) was extracted from the AFM scan profile. Subsequently, we plotted all those scanline profiles on the same graph (Figure 3), showing that the scanline profiles correspond to the real geometry of the calibration grating as imaged by SEM [17].

**Figure 4:**
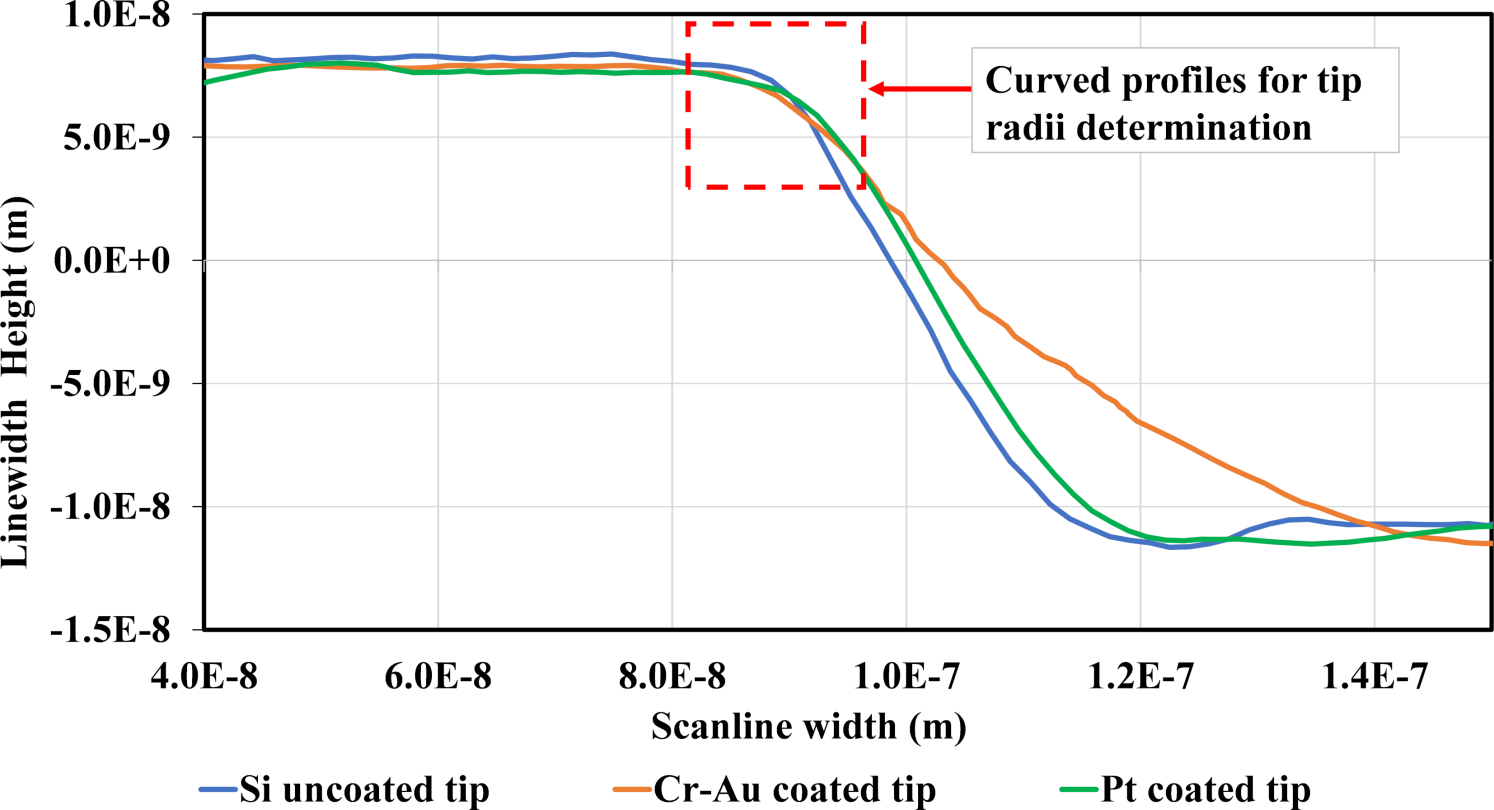
Comparison of the AFM scanline profiles of all tips scanning across the corner edge of the 20 nm calibration grating yielding the curves profiles for tip radius determination.

The lines in the lower panel of Figure 3 were created through connecting the point-to-point data of the AFM tip position while the tip was moved along the calibration grate. The height of the lines indicates the height of the grate. At this point, we were interested in tracing the curved profiles at the edge corners of the height grate. Hence, we zoomed in that area and scanned each tip again in a smaller scan area covering the grate corner with the same scanning process and load conditions. According to a graphical structure of the HS-20MG height calibration standard purchased from Budget Sensor [15], the grate height pattern made of silicon oxide (SiO_2_) has a height of 20 nm, a grate distance of 2 µm, and a pitch distance of 5 µm on top of the silicon substrate. So, the height distance that the tip end can slide along is equal to the distance measured from the corner edge hit point to the apex of the tip end on the slope cone surface of the tip (see Figure 2), which is longer than the 20 nm grate height. It is perceived that any cone-shaped tip (40° cone angle) with a radius greater than 8 nm can slide over this edge corner properly. The three tips can keep contact with the grate surface during the scan because the top surface of the grate height is 3 µm, allowing the tips with a radius greater than 8 nm to slide along it. Additionally, the scan rate is set at 0.03 Hz for an 800 nm scan length, that is, the scan velocity of the tip is 54 nm/s (scan rate × scan length × 2.25 = 54 nm/s), which is appropriate for the three different tips. The resulting height images for all tips (see Supporting Information File 1, Section S2) are in accordance with the analytical model of tip–sidewall contact in sliding motion as denoted in Figure 2. Namely, the bright area means that the tip apex physically touched the grate’s curved surface of the corner edge. In contrast, the dark area represents inability of the tip to reach the bottom corner of the grating structure (a 20 nm SiO_2_ column [15]), because the tip cone angle is not parallel to the tip cone surface. The scanline profiles of all tips were extracted from the height images and compared in the same plot to ascertain the arc section for determining the tip radii with the curve fitting method (Figure 4). We took the data points of tip position from each curve as marked with the red dash line in Figure 4 and then determined the radius of the tip by fitting the arc curves using a nonlinear regression function as described in the next section.

### Curve fitting with nonlinear regression

**Figure 5:**
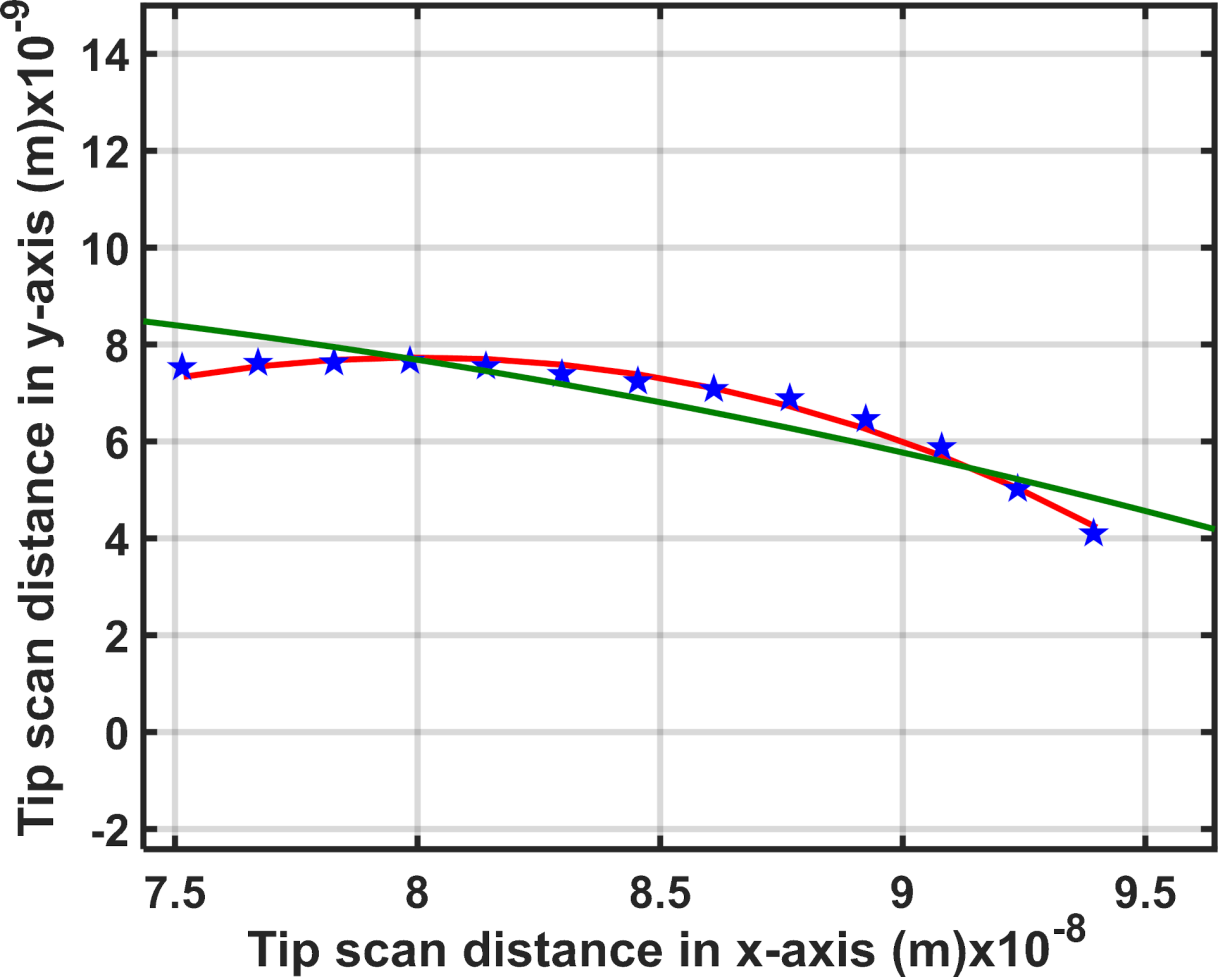
A typical curve fit to the measured data points of the Pt-coated tip (blue stars) with the nonlinear regression function fit (red curve) and the least-mean square fit (green curve).

Since the data points of tip position in the scan profile (Figure 4) form an arc curve, these data points are suitable to be fitted with a nonlinear regression method. The data points of tip position (the measured displacement of the scanning tip) in (*x*, *y*) coordinates were imported into MATLAB. Then, we used a nonlinear regression function in MATLAB to fit the arc curve to the measured data to estimate the tip radii. The nonlinear regression function of a circular curve on the *x*–*y* plane, *y* = (*x*_1_^2^ + *x*_2_^2^ + *b*_1_*x*_1_ + *b*_2_*x*_2_ + *b*_3_) was used for fitting the circular curve (the red curve in Figure 5) to the measured data on the Cartesian *x*_1_ and *x*_2_ axes (blue stars in Figure 5, typical data of the uncoated Si tip) and finding the center of the arc. The parameters *b*_1_, *b*_2_, and *b*_3_ are vector parameters. Then, the mean distance (*X*_m_ and *Y*_m_) from the center to those data points on that arc was estimated by the function. Finally, the tip radius can be calculated by *R* = ((*X*_m_^2^ + *Y*_m_^2^) – *B*_3_)^1/2^, where *B*_3_ is an error term (see Supporting Information File 1, Section S3). We also used the least-mean square fitting method to fit the measured data for the curvature radius. The least-mean square equation is (*x*, *y*)*L* = [(*x*^2^ − *d_x_*) + (*y*^2^ − *d_y_*)]/2; then the curvature radius of the tip can be calculated by 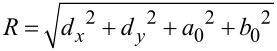, where *x* and *y* are the measured displacement values of the AFM tip in *x* and *y* directions, *d_x_* and *d_y_* are the mean distances from the center of the circle in *x* and *y* directions, and *a*_0_ and *b*_0_ are constants obtained from the least-mean square solution (see more details in Supporting Information File 1, Section S3). The fit results are shown in Figure 5. It is found that the nonlinear regression function gives a better fit (RMSE = 8.41 × 10^−18^, red curve) than the least-mean square method (RMSE = 4.60 × 10^−10^, green curve). Therefore, we chose the nonlinear regression function to determine the curvature radii of all tips.

## Result and Discussion

### Nonlinear regression curve fit

Fitting all obtained tip scan profile curves yielded the following estimated values of the tip radii. The radius of the uncoated Si tip is approx. 10.54 nm, which is smaller than the radius of the Pt-coated tip (approx. 25.78 nm) and the Cr/Au-coated tip (approx. 29.61 nm). The latter two values are consistent with the standard nominal radii specified by the manufacturer. This shows that the nonlinear regression method gives an accurate estimate because the values of root mean squared error (RMSE) from the fitting results are very small, that is, 2.67 × 10^−18^ for the uncoated Si tip, 3.43 × 10^−18^ for the Pt-coated tip, and 4.82 × 10^−18^ for the Cr/Au-coated tip. The R-squared values of all fitting results are infinity since R-squared is not valid for nonlinear regression models. However, there might have been a few errors in the scanline of the Cr/Au-coated tip that moved up a bit more in the approach regime (Figure 4) while sliding toward the sidewall of the grate height. Possibly, some particles stuck to the tip surface because of contaminations on the grating, resulting in a relatively high RMSE. Also, there might be some errors in the estimated value of the uncoated Si tip (approx. 10.54 mm, which is greater than the nominal radius provided by the tip manufacturer) caused by the uncertainty of the quality of the corner edge surface of the grate and variations of the edge radius. Some areas of the edge might have radii greater than or less than 2 nm, which are very difficult to measure. Researchers who want to calibrate the tip radius before the AFM experiment need to perform a thorough cleaning of the AFM tip and a characterization of the edge radius of the standard grate before measuring the radius of the AFM tip.

### Thorough measurement and distribution of the determined tip radius

Scanned height images of the standard grating (uncoated Si tip, Pt-coated tip, and Cr/Au-coated tip) were taken to extract the cross section for fifteen profiles of the tip radius, which are the curved arc profiles over the corner edge of the grate (see Supporting Information File 1, Section S4). To estimate the curvature radius of the tips, the data points of the AFM tip positions of each of the fifteen curve profiles were imported to MATLAB. Then, a nonlinear regression with the circle function was fit to those data points to find the center of the arc, thereby getting the curvature radius of the AFM tip. The fifteen determined radius values of each tip (uncoated Si tip, Pt-coated tip, and Cr/Au-coated tip) obtained from independent cross-section profiles were plotted in distribution graphs (see Figure S4-5, Figure S4-6, and Figure S4-7 in Supporting Information File 1, Section S4). It was found that the measured values of three AFM tips are fairly normally distributed around the mean values (10.27 ± 0.095 nm (standard error) for the Si tip radius, 25.76 ± 0.08 nm (standard error) for the Pt-coated tip radius, and 29.67 ± 0.18 nm (standard error) for the Cr/Au-coated tip radius) with standard deviations (STDs) of 0.33, 0.28, and 0.70 nm, respectively.

## Conclusion

Three different AFM tips underwent actual tip radius measurements using an in situ probe characterizer (HS-20MG Height Calibration Grating Standard) under sub-nanonewton scanning load. The curvature radius of each tip was estimated by fitting the measured data points of the tip position on the curved scanline at the edge corner of the grating structure with a nonlinear regression function. The results have shown that the tip radii obtained from reproducible in situ measurements fit with a nonlinear regression function are in the range of the nominal values (<30 nm of the Pt-coated tip, <35 nm of the Cr/Au-coated tip) given by the manufacturer, except for the radius of the uncoated Si tip, which is larger than the nominal value by about 2.27 nm. The curved profiles of both coated and uncoated tips show similar curved profiles, but different curvature radii. The curve fitting results indicated that our estimation model is reasonably accurate. However, more accuracy could be achieved if we had more data points of the AFM tip position in the curved scan profile for nonlinear regression curve fitting.

## Supporting Information

Section S1: Height images of the scanned specimens of calibration standard grating within 10 µm × 10 µm. Section S2: Height images of the scanned corners of the calibration gate at smaller areas. Section S3: Nonlinear regression function used for determining the tip radii.

File 1 Additional experimental data.
