# Supplementary material for: Determination of the radii of coated and uncoated silicon AFM sharp tips using a height calibration standard grating and a nonlinear regression function
Source: Beilstein J Nanotechnol. 2023 Dec 15;14:1200–7. doi: 10.3762/bjnano.14.99 (PMC10729132; doi:10.3762/bjnano.14.99)
Supplement: File 1 — Additional experimental data. [file Beilstein_J_Nanotechnol-14-1200-s001.pdf]

BEILSTEIN JOURNAL OF NANOTECHNOLOGY

Supporting Information

for

Determination of the radii of coated and uncoated silicon AFM sharp tips using a height calibration standard grating and a nonlinear regression function

Perawat Boonpuek and Jonathan R. Felts

Beilstein J. Nanotechnol. **2023,** *14,* 1200–1207. doi:10.3762/bjnano.14.99

Additional experimental data

License and Terms: This is a supporting information file under the terms of the Creative Commons Attribution License (https://creativecommons.org/licenses/by/4.0). Please note that the reuse, redistribution and reproduction in particular requires that the author(s) and source are credited and that individual graphics may be subject to special legal provisions.
The license is subject to the *Beilstein Journal of Nanotechnology* terms and conditions: (https://www.beilstein-journals.org/bjnano/terms)

S1: Height images of the scanned specimens of calibration standard grating within 10 µm × 10 µm

Figure S1 shows top views of the height images of calibration standard grating scanned by the AFM tips from right to left (as shown in Figure 3 in the main manuscript):

Si uncoated tip

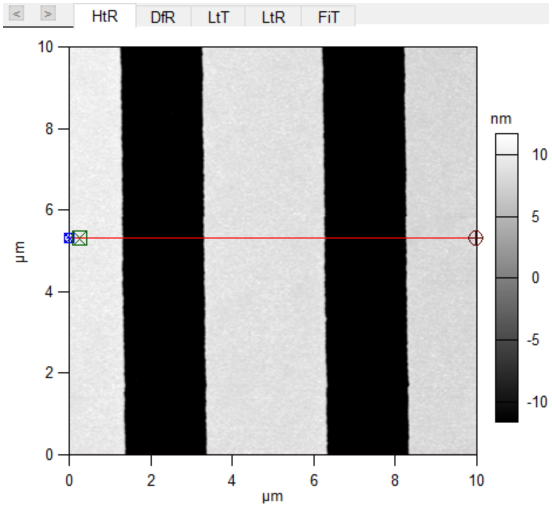

Pt coated tip

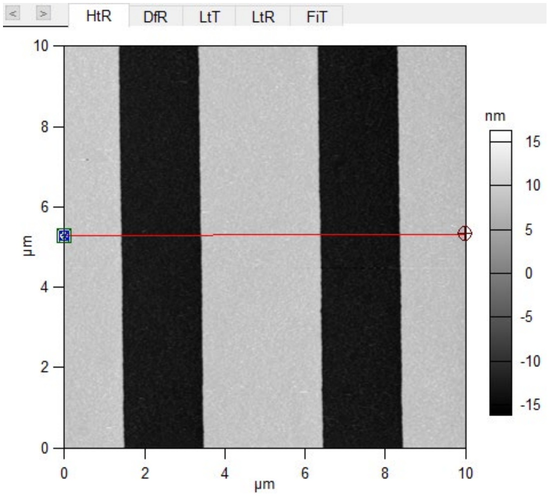

Au coated tip

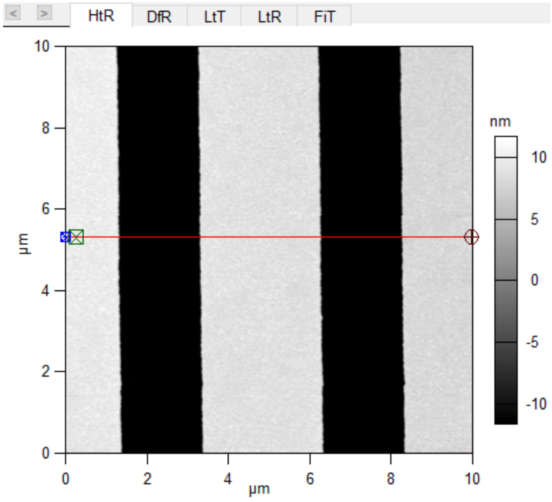

Figure S1: Height images of the scanned specimens of calibration standard grating by three different tips.

S2: Height images of the scanned corners of the calibration gate at smaller areas

Each of these images shows the height image at the smaller scanned area and was measured for the profiles of the grating structure scanned by the AFM tip from right to left (as shown in Figure 3 in Manuscript). The red cross-section lines were used to extract the profiles of the single local grating structure to determine the real radius of the tip through curve fitting with a nonlinear regression function.

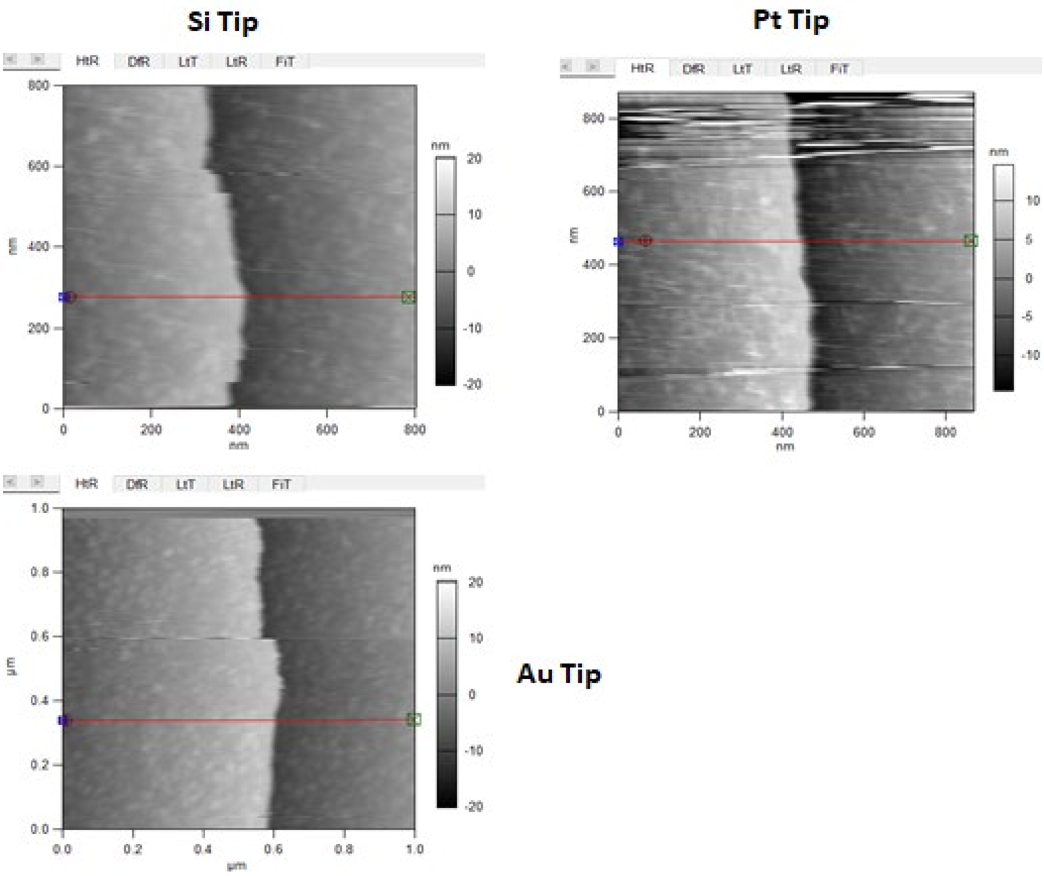

Figure S2: Scanned images of a smaller area at the edge corner of the standard grating.

Note: When we measured the scan profiles along the red line at different cross-section areas of the scanned image and took the average, we found large deviations of the estimated radius from the radius measured by the single cross-section redline.

S3: Nonlinear regression function used for determining the tip radius

Nonlinear Regression function: y = (x1^2 + x2^2 + b1*x1 + b2*x2 + b3) using MATLAB.

Method: Fitting of x1 and x2 data to the circular curve created by the nonlinear regression function to find the center of the arc of the measured data points. Then, find the mean distance (Xm & Ym) from the center to those data arc curves. The outcome is tip radius: R = sqrt((Xm^2 + Ym^2)-B(3)). We also used the least-mean square to fit the same measured data points for the curvature radius of the tip compared to the fitting result from the nonlinear regression function. See MATLAB code below.

MATLAB code using both the Nonlinear Regression function and the Least-mean square fit

```
%X0 = CrossSectionHeightProfileForRadiusCalibration(:,1:2);

%X = X0;

X = ArcProfilePtTip; % this is the scan curve profile data of the coated Pt tip for example.

% Nonlinear regression function fitiing

circlefun = @(b,X) (X(:,1).^2 + X(:,2).^2 + b(1)*X(:,1) + b(2)*X(:,2) + b(3));

y = zeros(length(X(:,1)),1);

beta0 = [0 0 400];

%Fit the measured data to nonlinear regression function model.

mdl = fitnlm(X,y_1,circlefun, beta0);

B = mdl.Coefficients.Estimate;

Xm = -B(1)/2;

Ym = -B(2)/2;

R_nl = sqrt((Xm^2 + Ym^2) - B(3));

A = atan2(X(:,2)-Ym, X(:,1)-Xm);

Ycir = R_nl*sin(A) + Ym;

Xcir = R_nl*cos(A) + Xm;


% Least-mean square fitting

X0 = ArcProfilePtTip; % import the measured data of tip scan profile for each type of AFM tip

x = X0(:,1);

y = X0(:,2);

mx = mean(x); % get mean of tip position in x-axis

my = mean(y); % get mean of tip position in y-axis

X_ls = x-mx ; % differences from means

Y_ls = y-my ; % differences from means
```

```
dx2 = mean(X_ls.^2);

dy2 = mean(Y_ls.^2); % variances

L = [x,y] \ (x.^2 - dx2 + y.^2 - dy2)/2; % Solve least mean squares problem

a0 = L(1);

b0 = L(2);  % L is the 2 x 1 solution array [a0;b0]

R_ls = sqrt(dx2 + dy2 + a0^2 + b0^2); % Calculate the radius

a = a0 ;

b = b0 ; % Locate the center of circle

R0 = sqrt(((x-a).^2) + (y-b).^2);

RMSE2 = sqrt(mean((R_ls - R0).^2));

th = (75:0.1:83)*(pi/180);

xunit = R_ls*cos(th) + a;

yunit = R_ls*sin(th) + b;

% Plot both results on the same graph

figure

plot(X(:,1), X(:,2), 'p')

hold on

plot(Xcir, Ycir, '-r', 'LineWidth',2);

hold on

plot(x, y, 'p')

plot(xunit, yunit, '-b', 'LineWidth',2);

hold off

grid

axis('equal')
```

As a result, the nonlinear regression function gives the best fit when compared to the least-mean square method as shown in the figure below. Note: Blue stars are the measured data points. The red curve is the nonlinear regression fit. The green curve is the least-mean square fit.

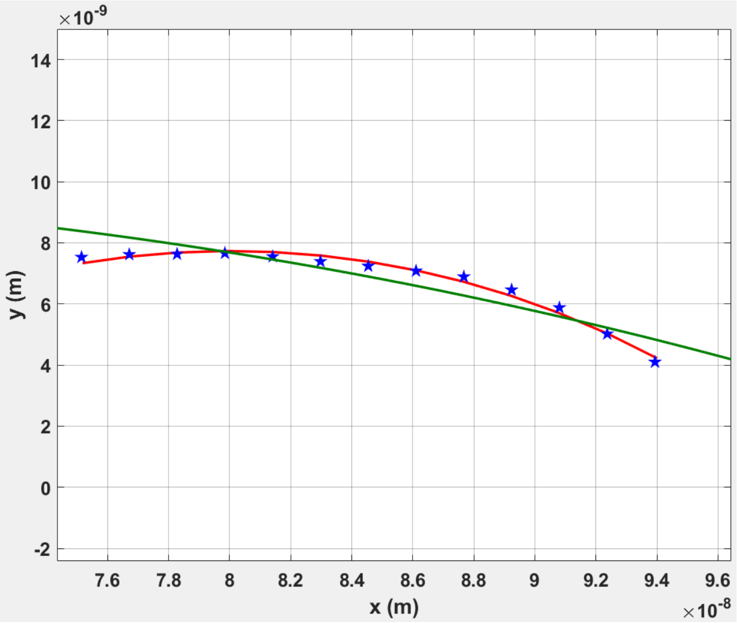

- Red curve is the nonlinear regression fit.
  Root Mean Squared Error (RMSE): 8.418e-18
  R-Squared: -Inf
- Green curve is the least-mean square fit.
  Root Mean Squared Error (RMSE): 4.60e-10
  R-Squared: -Inf

Figure S3-1: Comparison of curve fitting results between nonlinear regression function and least-mean square method.

The curve fitting results from using only the best fitting model, that is, the nonlinear regression function, are shown below. Note: Blue stars are the measured data points. The red curve is the nonlinear regression fit.

Si tip

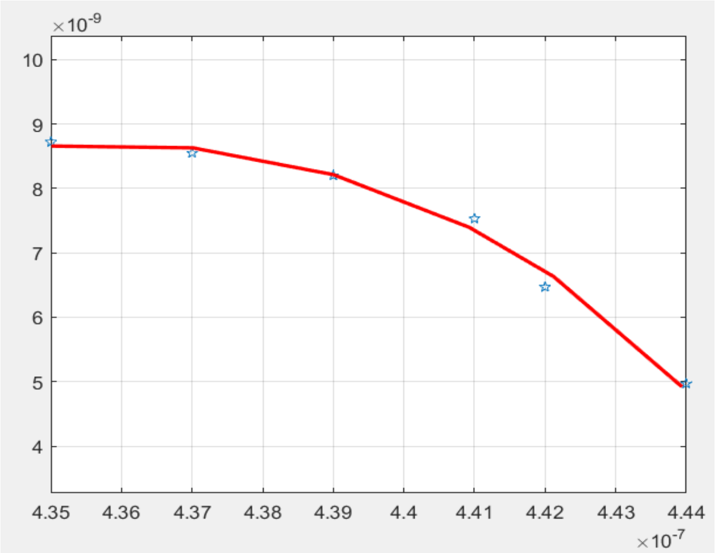

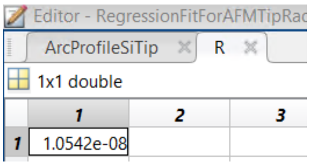

Root Mean Squared Error: 2.67e-18
R-Squared: -Inf

Figure S3-2: Curve fitting result using nonlinear regression function for the uncoated Si tip.

Pt tip

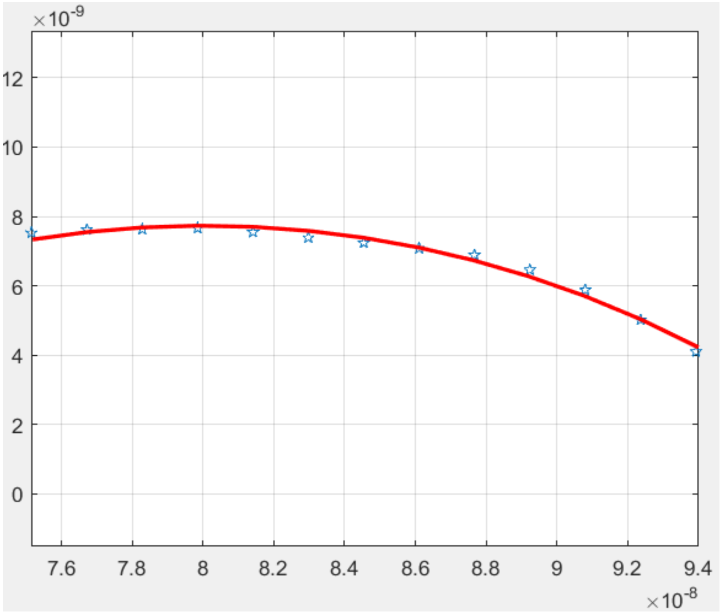

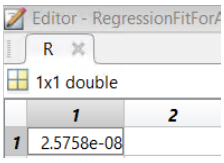

Root Mean Squared Error: 3.43e-18
R-Squared: -Inf

Figure S3-3: Curve fitting result using nonlinear regression function for the Pt-coated tip.

Au tip

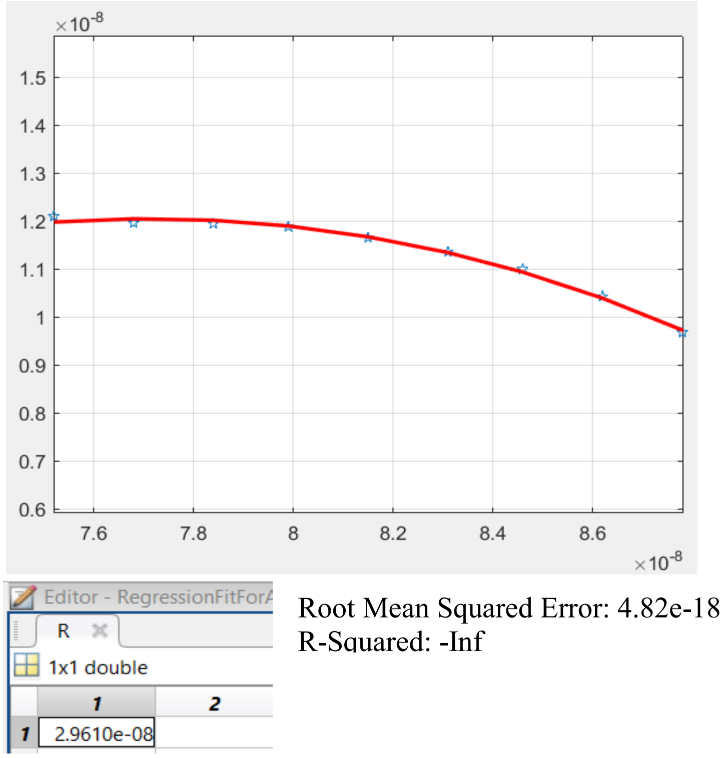

Figure S3-4: Curve fitting result using Nonlinear regression function for the Au-coated tip.

S4: Reproducibility of measurement method on independent scanlines of the same scanned images for three tips and distributions of the determined radius values

Each of the scanned height images of the standard grating by there tips (uncoated Si tip, Pt-coated tip, and Au-coated tip) was taken to measure the cross section for 15 profiles of the tip radius which are the arc curved profiles over the corner edge of the grate. See the following figures:

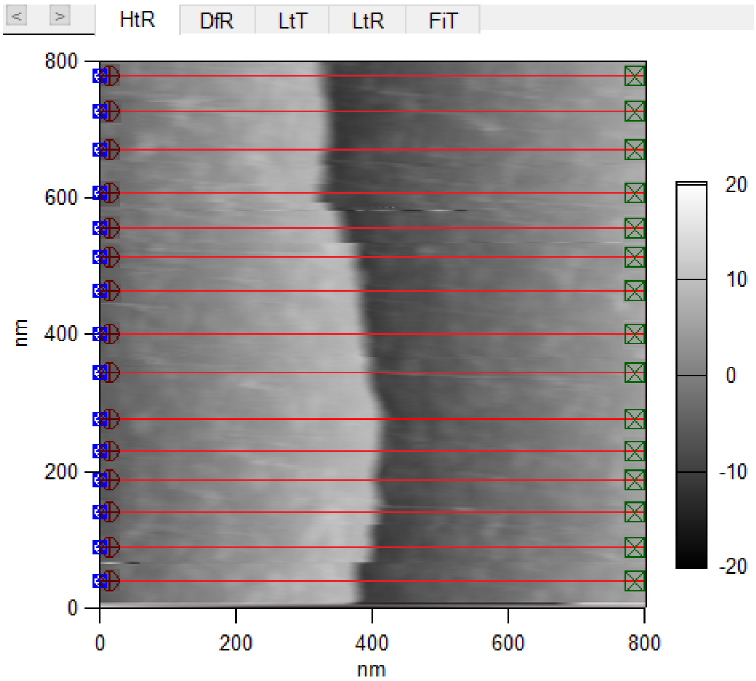

Figure S4-1: 15 measured radius profiles of the uncoated Si tip on different cross section lines.

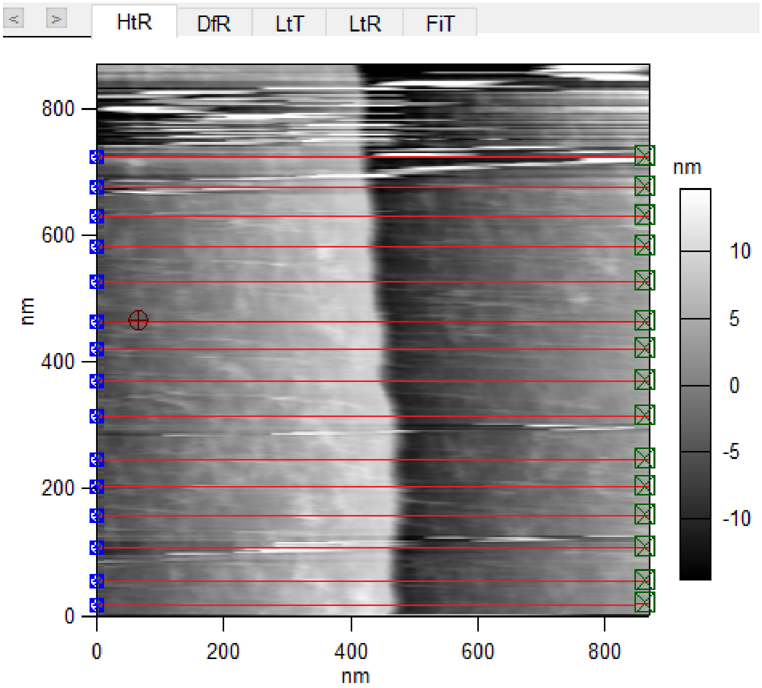

Figure S4-2: 15 measured radius profiles of the Pt-coated tip on different cross section lines.

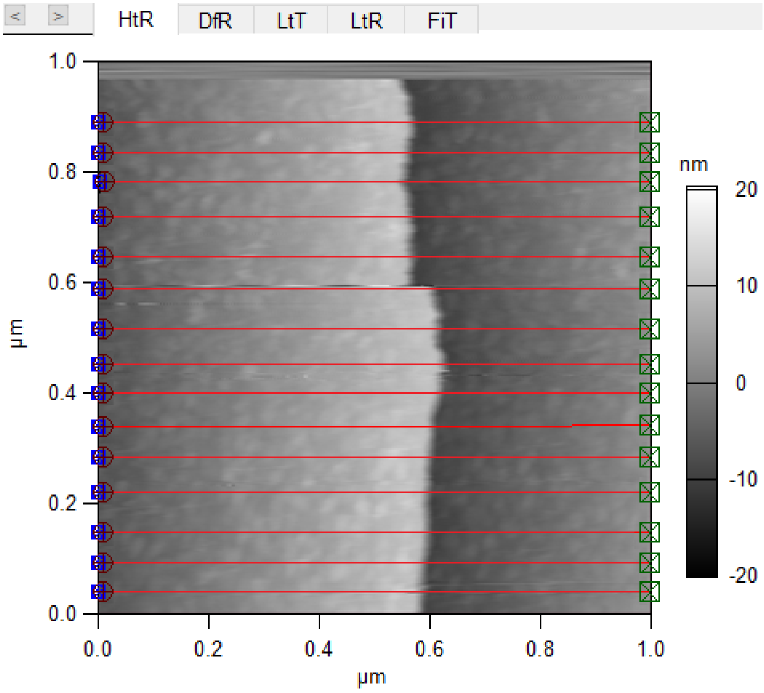

Figure S4-3: 15 measured five radius profiles of the Cr/Au-coated tip on different cross section lines.

Since there is a lot of measured profiles extracted from three images above, we take the average of coordinate values of 15 scanned profiles of each tip to make a single readable graph as shown in Figure S4-4 below.

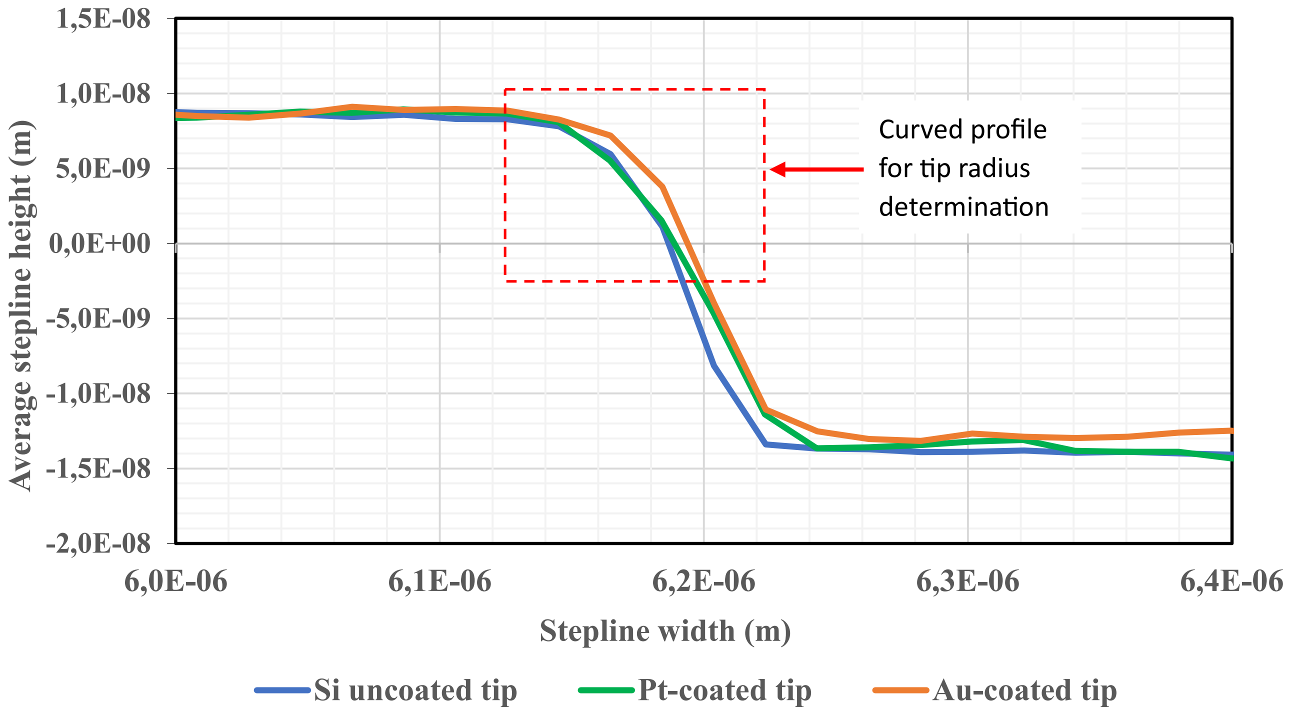

Figure S4-4: Three scanned profiles of the averages of AFM tip positions taken from 15 scanned profiles of each tip.

To estimate the curvature radius of all tips, the data points of AFM tip positions on each of fifteen curved profiles were imported to MATLAB. Then, the nonlinear regression function with the circle function for finding the center of the arc was fit to those data points, thereby getting the curvature radius of the AFM tip. The 15 values of the determined radius of each tip (Si uncoated tip, Pt-coated tip, and Cr/Au-coated tip) obtained from independent cross-section profiles were plotted on distribution graphs as shown in Figure S4-5, Figure S4-6, and Figure S4-7 below. The bar graphs in the distribution plots show the number of measured values of the AFM tip radii (frequency on y axis) lie in the range of the measured radius on x axis. The distribution curve in each graph was created by calculating the distribution values using the measured radius, mean and standard deviation.

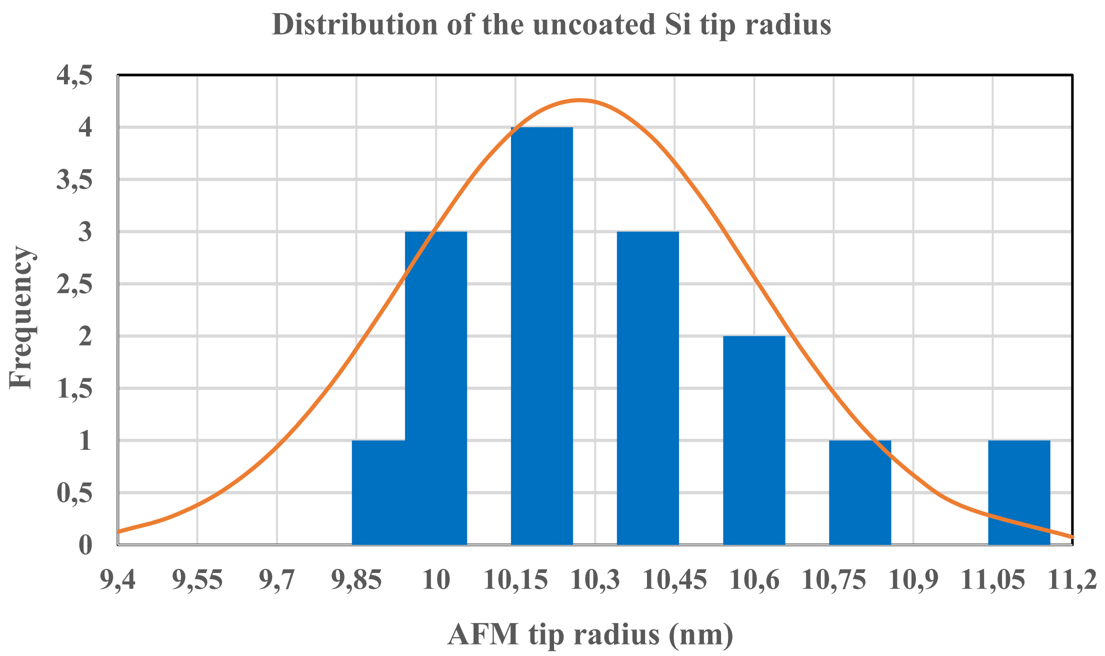

Figure S4-5: Normal distribution of 15 values of Si tip radius measured at different sections of the standard grating, mean = 10.27 nm, standard deviation = 0.33 nm, standard error = 0.095 nm.

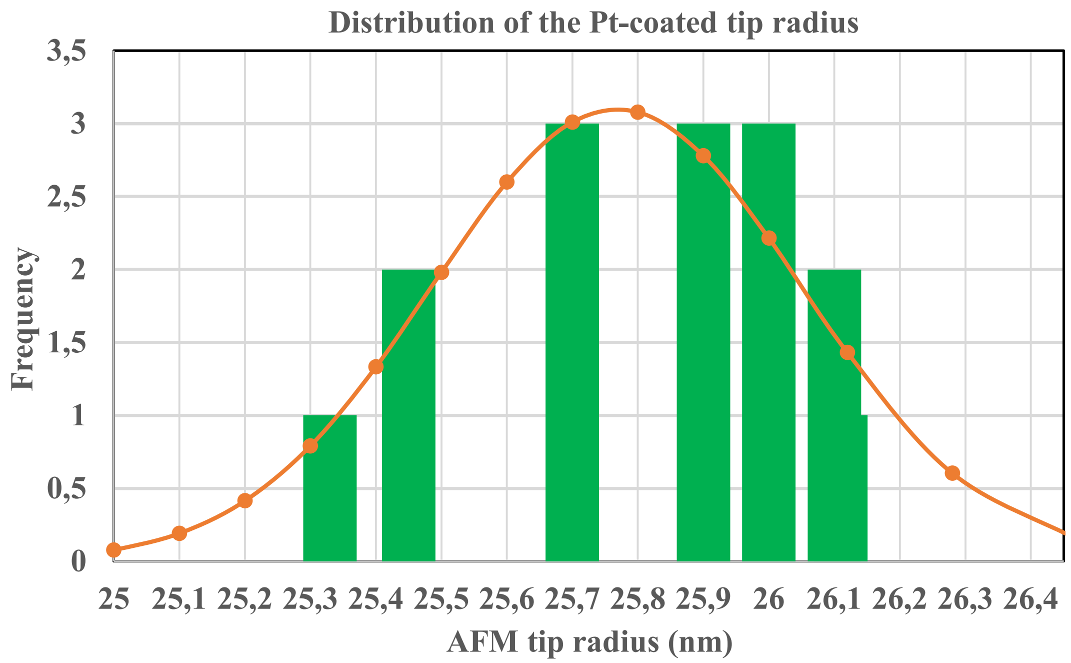

Figure S4-6: Normal distribution of 15 values of Pt-coated tip radius measured at different sections of the standard grating, mean = 25.76 nm, standard deviation = 0.32 nm, standard error = 0.082 nm.

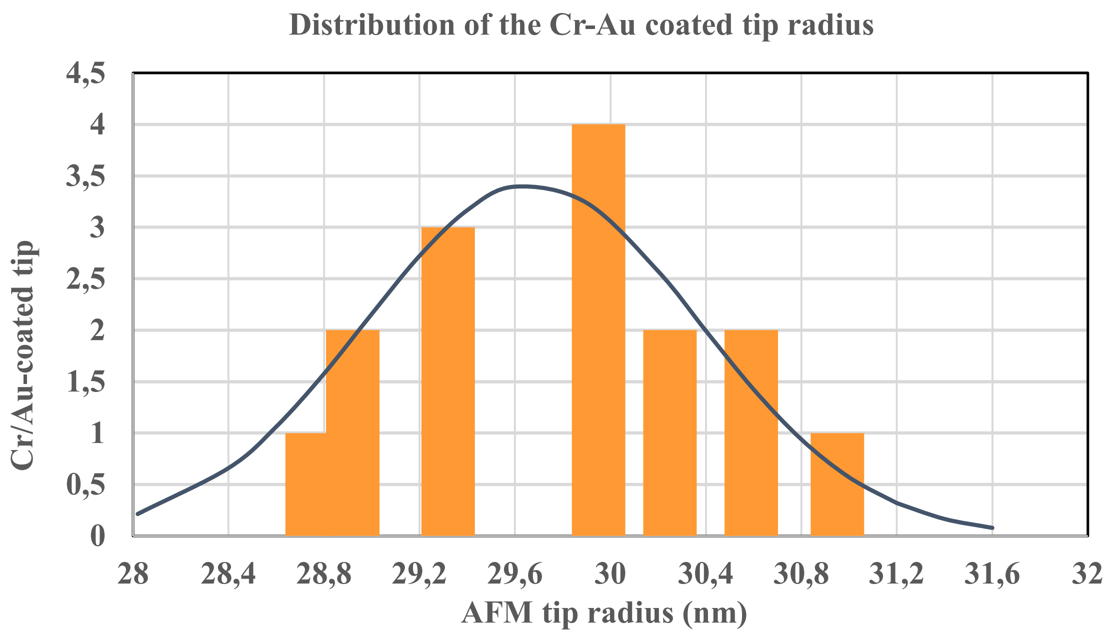

Figure S4-7: Normal distribution of 15 values of Cr/Au-coated tip radius measured at different sections of the standard grating, mean = 29.67 nm, standard deviation = 0.70 nm, standard error = 0.18 nm.

It is found that the measured values of three types of the AFM tip fairly lie in normal distribution around the mean with standard deviations (STDs) of 0.33, 0.28, and 0.70 nm, respectively.
